# Supplementary figures and images for: Maximal Reduction of STIC Acquisition Time for Volumetric Assessment of the Fetal Heart—Benefits and Limitations of Semiautomatic Fetal Intelligent Navigation Echocardiography (FINE) Static Mode
Source: J Clin Med. 2022 Jul 14;11(14):4062. doi: 10.3390/jcm11144062 (PMC9320472; doi:10.3390/jcm11144062)

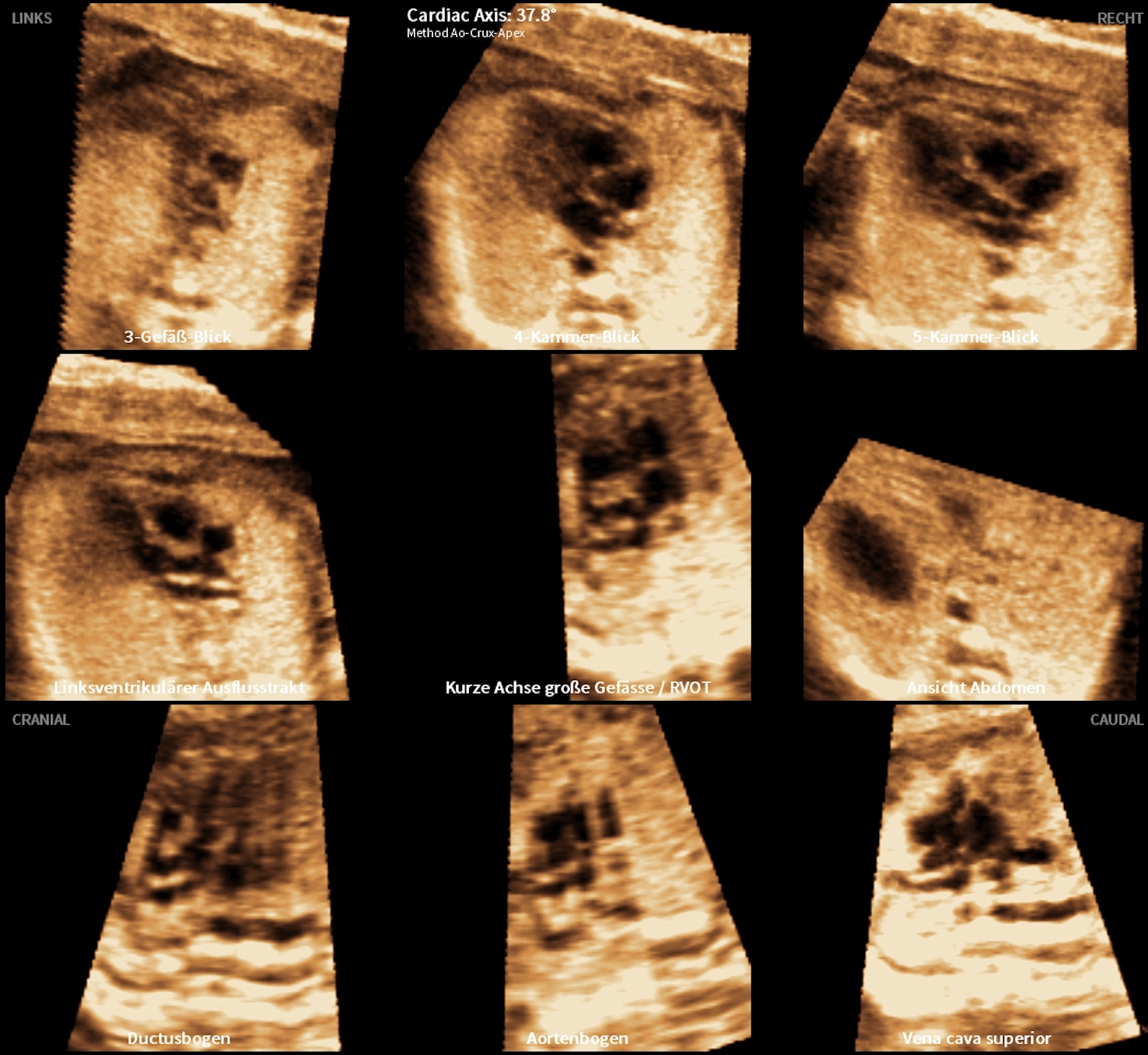

Supplement: Supplementary file 1 [file jcm-11-04062-s001.zip › FINE Standard Mode Moving Fetus Picture.png]

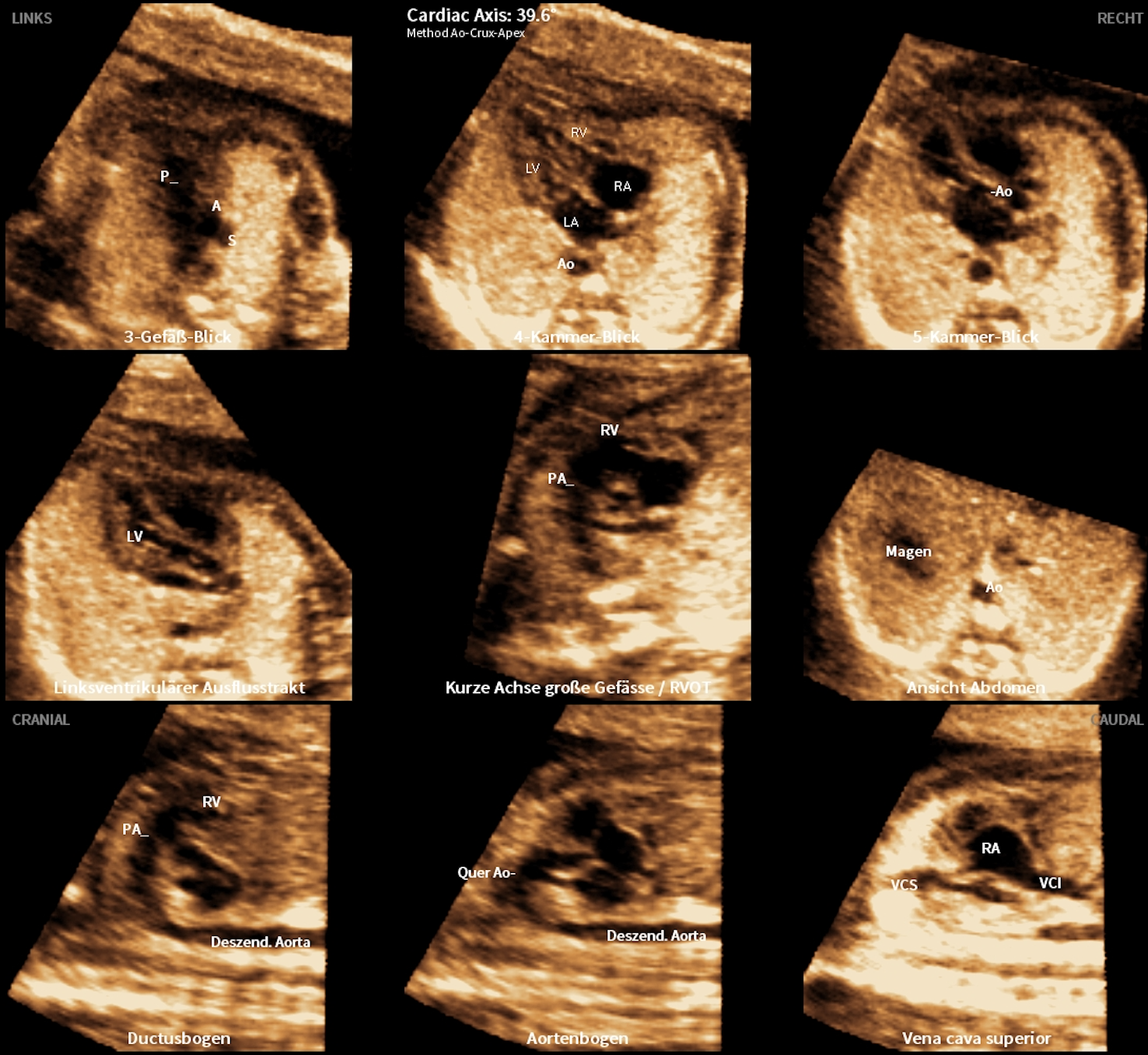

Supplement: Supplementary file 1 [file jcm-11-04062-s001.zip › FINE Static Mode Moving Fetus Picture.png]
